# Consumers’ experiences and perspectives on reporting adverse drug reactions using digital tools: a qualitative study

**DOI:** 10.1007/s11096-026-02193-1

**Published:** 2026-07-22

**Authors:** Mohammed Gebre Dedefo, Gizat M. Kassie, Eyob Alemayehu Gebreyohannes, Renly Lim, Elizabeth Roughead, Lisa Kalisch Ellett

**Affiliations:** 1 Quality Use of Medicines and Pharmacy Research Centre, College of Health, Adelaide University, Adelaide, Australia; 2 South Australian Health and Medical Research Institute, Adelaide, SA Australia; 3 College of Medicine and Public Health, Flinders University, Adelaide, SA Australia; 4 Centre for Optimisation of Medicines, School of Allied Health, The University of Western Australia, Perth, Australia; 5 Centre for Translational Research, Institute for Research, Development and Innovation, IMU University, Kuala Lumpur, Malaysia

**Keywords:** Drug-related side effects and adverse reactions, Digital technology, Pharmacovigilance, Adverse drug reaction reporting systems, Models (Theoretical)

## Abstract

**Introduction:**

Spontaneous adverse drug reactions (ADRs) are underreported. Digital reporting systems are increasingly used to address this issue; however, limited knowledge exists about the barriers and facilitators influencing their use by consumers.

**Aim:**

To explore facilitators and barriers influencing consumers’ use of digital tools for ADR reporting, and to identify the factors, expectations and preferences that affect their engagement with these tools.

**Method:**

An exploratory qualitative descriptive study using semi-structured in-depth interviews was conducted among Australian adults who self-reported having experienced an ADR. Interviews were conducted face-to-face or via Zoom, audio-recorded, transcribed verbatim, and analysed inductively using reflexive thematic analysis. Themes were mapped onto domains of the Combined Technology Acceptance Model and Theory of Planned Behaviour (C-TAM-TPB) framework.

**Results:**

Fourteen participants were interviewed. Consumers expressed positive intentions to use online ADR reporting tools. Identified facilitators included user-friendly and accessible tools that are simple to complete, the use of images to aid comprehension, integration with existing patient health records, the ability to submit reports to both healthcare professionals and regulators, and a tool designed specifically for consumers using layperson language. Identified barriers included lack of awareness that consumers could report ADRs using online tools, the need for registration, complex medical terminology, compulsory fields, and the absence of feedback after submission.

**Conclusion:**

Consumers were willing to use online ADR reporting tools but were largely unaware of their existence. Enhancing usability through user-friendly design, and reporting forms specifically designed for consumers may increase engagement. Addressing barriers such as lack of awareness, registration requirements, medical jargon, and lack of feedback may improve usability and contribute to strengthening pharmacovigilance systems.

**Supplementary Information:**

The online version contains supplementary material available at https://doi.org/10.1007/s11096-026-02193-1.

## Impact statements

- Consumers expressed a high willingness to use digital adverse drug reaction (ADR) reporting tools.
- Consumers indicated that the use of digital ADR reporting tools would increase if the tools were user-friendly, designed using lay language, integrated with existing patient health records, enabled reports to be shared with both healthcare professionals and regulators and provided acknowledgment and feedback from regulators.
- To maximise the use of digital ADR reporting tools, barriers such as lack of awareness, complex medical terminology, privacy concerns, the absence of feedback after ADR submission, and compulsory fields in ADR reporting forms that hinder consumers from fully completing them need to be addressed.

## Introduction

Spontaneous adverse drug reaction (ADR) reporting is a fundamental component of pharmacovigilance [1, 2] and can detect new, rare and serious ADRs that were not identified during pre-marketing clinical trials [2, 3]. Spontaneous reporting captures real-world data from diverse patient populations, offering insights into medicine safety in routine clinical practice [4]. However, underreporting is a major limitation [5–7], influenced by factors such as insufficient awareness of ADR reporting methods, uncertainty about whether a reaction is medicine-related, perceptions that common ADRs don’t require reporting and time constraints experienced by clinicians [2–4, 8, 9]. Systematic reviews report that consumers find ADR reporting time-consuming and that they would be more likely to report if the process were simplified [9, 10].

Digital tools like mobile apps and web-based systems are increasingly used to address underreporting in pharmacovigilance systems [11, 12] and to improve the rate and quality of ADR reporting [11, 13–15]. For example, ADR reporting by consumers and healthcare professionals (HCPs) in the UK increased by 83.7%, from 30,025 reports in the year prior to the launch of the Yellow Card Scheme app in 2015 to 55,159 in the year following the launch of the Yellow Card Scheme app [11]. Similarly, in Nigeria the total number of ADR reports submitted by consumers increased from 3% (n = 55 of 2032 reports submitted) in the year prior to introduction of the Med Safety app for consumers to 18% (n = 627 of 3562 reports) in the year after [16]. Almost all (97%) reports submitted via the Med Safety app had a completeness score above 70%, allowing causality assessment of reported ADRs with suspected medicines [16]. Similarly, prior research highlights the importance of consumer-centred ADR reporting designs [17], with higher reporting rates observed in countries that implement consumer-centred reporting tools compared to those that do not [18]. These results indicate that digital technologies can increase the rate of ADR reporting and thereby strengthen pharmacovigilance systems.

While existing research has primarily focused on evaluating reporting rates and data quality associated with digital ADR reporting tools, limited evidence exists on consumers’ perspectives on the usability and design of these tools. There is also limited evidence on the specific features that consumers prioritise, as well as the barriers and facilitators influencing their engagement with digital ADR reporting systems. This gap is evident in a 2023 survey of Australian consumers, which indicated that only 12% had used the online ADR reporting website developed by the national medicines regulator [19], despite 80% expressing an intention to use digital tools for reporting [20]. This discrepancy indicates the presence of barriers related to the use of existing ADR reporting tools and suggests that these tools may not adequately align with consumer needs and preferences.

## Aim

This study aimed to investigate barriers and facilitators to reporting ADRs by consumers, to identify factors affecting the use of digital tools for ADR reporting by consumers, and to identify the expectations and preferences of consumers regarding the use of digital tools for reporting ADRs.

## Method

### Study design and development of the interview guide

An exploratory qualitative descriptive study was conducted using semi-structured in-depth interviews. This methodological approach was selected because it offers a comprehensive and precise depiction of the phenomenon being investigated [21, 22], and allows for the acquisition of substantial, descriptive data from participants, providing a thorough comprehension of their experiences and perspectives [21, 22]. The Consolidated Criteria for Reporting Qualitative Research (COREQ) checklist was used to ensure the quality of reporting [23].

An interview guide was developed based on a literature review and the findings from our previous survey [19, 20]. The full interview guide is provided in Supplementary file. Questions were designed to elicit detailed insights into consumers’ (members of the general public who use medicines) experiences with ADR reporting, their utilisation of digital tools for ADR reporting, their views on digital tools, expectations and preferences and potential barriers to using digital ADR reporting tools. The interviewer walked participants through the online ADR reporting form developed by Australia’s national medicines regulator as an example. The WHO recommends the important information required for causality assessment associated with spontaneous ADR reports, which includes information relating to the patient (sex, age, medical history), adverse event (severity, start date, treatment, and outcome), suspected medicine (name, dose, route of administration, start and stop dates, and indication for use), concomitant medications, risk factors, and reporter type (consumer or HCP) [3]. The online ADR reporting form selected as the example for this study included this information. Using the tool as a guide, we explored whether the terminologies were clear. Participants were asked to identify potential barriers to using such tools, as well as features they found helpful. Additionally, they were asked to share their suggestions to make such tools more accessible and user-friendly for reporting ADRs.

Prior to the commencement of the study, the interviewer conducted mock interviews with the research team and conducted a pilot interview with one consumer who was not included in the final analysis to test the interview guide and process. Subsequently, amendments were made to the interview guide and process based on the feedback received.

### Participants’ eligibility, recruitment process and data collection

Participants who completed our previous survey [19] were invited to indicate their interest in participating in a semi-structured in-depth interview. Participants who expressed interest were subsequently formally invited to participate in the interview via email. Additionally, participants in our research network were invited through direct email contact. Adults aged 18 years or older living in Australia who self-reported having previously experienced an ADR, who could speak and understand English, and who could provide written informed consent were eligible to participate. Age was categorised into 18–44, 45–64, and ≥ 65 years to reflect different stages of adulthood and potential differences in familiarity with and engagement in digital technologies, which may influence the use of digital ADR reporting tools. All interviews were conducted by the same researcher (MGD), a male PhD candidate with prior research experience who had received training in conducting qualitative interviews. The participants included in the study were not acquainted with the interviewer before the interviews commenced.

The interviews, which lasted between 40 and 80 min, were conducted face-to-face or via Zoom between March and November 2024. Interviews were audio-recorded and transcribed verbatim using Microsoft Word’s Dictate and Transcribe features. The initial transcripts generated by the software were manually reviewed to ensure accuracy, and transcription errors identified were corrected prior to data analysis.

### Study sampling

The sample size for the semi-structured interviews was based on data saturation [24], following the two principles recommended by Francis et al. [25]. Data collection and analysis were conducted concurrently. An initial set of 10 interviews was analysed to identify emerging themes in line with the a priori principle. Additional interviews were then conducted and analysed iteratively. Saturation was considered achieved when no new themes emerged in three consecutive interviews, meeting the predefined stopping criterion [25].

### Rigour

We employed the four criteria outlined by Guba [26], which are credibility, transferability, dependability, and confirmability in order to ensure rigour. Credibility refers to the truth of the findings, while transferability signifies that the results can be applied to other contexts [26]. Dependability indicates that the findings are consistent and reproducible, and confirmability represents the neutrality or the extent to which the study’s outcomes are influenced by the respondents rather than the biases, motivations, or interests of the researcher [26]. Credibility was achieved by using analyst triangulation, where the first researcher (MGD) conducted the coding and analysis, and the second researcher (GMK) independently coded and analysed a 20% sample of the data [27, 28]. Discrepancies were discussed and resolved between the two researchers [27]. To ensure transferability, the presentation of results provided a thick description of the research context and participants, enabling others to assess the applicability of the findings to different settings [27]. Dependability and confirmability were ensured by maintaining a transparent and replicable audit trail of research activities through documentation of the rationale behind methodological choices and interpretations, as well as the definition of generated codes and themes. Additionally, debriefing was conducted among members of the research team to confirm that the findings accurately reflected the participants experiences and perspectives [27].

### Theoretical framework

The combined technology acceptance model and theory of planned behaviour (C-TAM-TPB) framework was used in the analysis. The framework integrates the cognitive approach of the Theory of Planned Behaviour (TPB) which focuses on individuals’ attitudes and beliefs toward new technology, and the Technology Acceptance Model (TAM) which assumes that users decide when and how they will use a new technology based on its perceived usefulness and ease-of-use [29]. We used the C-TAM-TPB framework because it provides a comprehensive framework for understanding the factors related to consumers experiences, attitudes, beliefs, and the use of technology that affect consumers’ behaviour when adopting new technology. The C-TAM-TPB framework comprises six domains of constructs, including perceived usefulness, ease of use, attitude, subjective norm, perceived behavioural control, and behavioural intention. These constructs are defined as follows [30]: attitude is “an individual's positive or negative feelings (evaluative affect) about performing the target behaviour”; subjective norms are “the person’s perception that most people who are important to him or her think he or she should or should not perform the behaviour in question”; perceived behavioural control is “the perceived ease or difficulty of performing the behaviour”; perceived usefulness is “the degree to which a person believes that using a particular system would enhance his or her job performance”; and ease of use is “the degree to which a person believes that using a particular system would be free of effort”.

### Data analysis

The analysis was conducted using reflexive thematic analysis [31]. Reflexive thematic analysis was used as a flexible yet systematic method to identify patterns and themes within qualitative data, enabling an in-depth interpretation of consumers’ experiences, perceptions and preferences regarding digital ADR reporting tools [31]. The analysis initially followed an inductive approach to generate codes and themes directly from the data, followed by a deductive step to map the findings to the selected framework to contextualise and interpret the results [31]. NVivo 14 software (QSR International Pty Ltd., Doncaster, Victoria, Australia) was used to facilitate the organisation, coding, management and thematic analysis of the qualitative interview data. Two researchers (MGD and GMK) independently generated codes after familiarising themselves with interview transcripts. They subsequently discussed their interpretations and coding to reflect on perspectives and refine the developing analysis. A third researcher (EAG) contributed to these discussions when differences in interpretation arose. After coding and analysing a 20% sample of the interview transcripts and reaching agreement on the initial codes, the remaining interviews were coded by one researcher (MGD) [28]. The research team comprised individuals with backgrounds in pharmacy and health research, all of whom had experience in conducting qualitative research. The team engaged in ongoing reflexive discussions throughout the analysis, critically reflecting on their assumptions and how these may have shaped coding and theme development. The initial themes were then generated, reviewed, defined and mapped onto the six domains of the C-TAM-TPB framework [30].

Finally, a report of the codes, themes, and de-identified participant quotes to illustrate the themes were produced by exporting the results from NVivo 14 to Microsoft Excel and Word documents. The initial codes, themes, and the quotes in the themes were shared with all the researchers, and all researchers participated in the overall interpretation and synthesis of the results.

### Ethics approval

Ethics approval to conduct this study was obtained from the University of South Australia Human Research Ethics Committee (Ethics protocol number: 204984) on November 2, 2023. This study adheres to the Australian National Statement on Ethical Conduct in Human Research (updated 2023) and the Declaration of Helsinki.

## Results

### Participants

Fourteen participants were interviewed. The majority were female (n = 9) and nearly half (n = 6) were aged 45 to 64. Five had completed a vocational education and training (VET) certificate, associate diploma, or graduate diploma, while nine held a bachelor’s degree or higher (Table 1). Most participants were from Victoria (n = 5) and South Australia (n = 5). The majority of interviews were conducted via Zoom (n = 12).

**Table 1:** Characteristics of the participants (N = 14)

Characteristics of the participants		Number
Sex	Female	9
	Male	5
Age	18–44	3
	45–64	6
	> 65	5
Highest level of education	VET* certificate, associate diploma, or graduate diploma	5
	Bachelor’s degree	5
	Higher than bachelor’s degree	4

*VET: Vocational Education and Training

### Themes

**Fig. 1:**
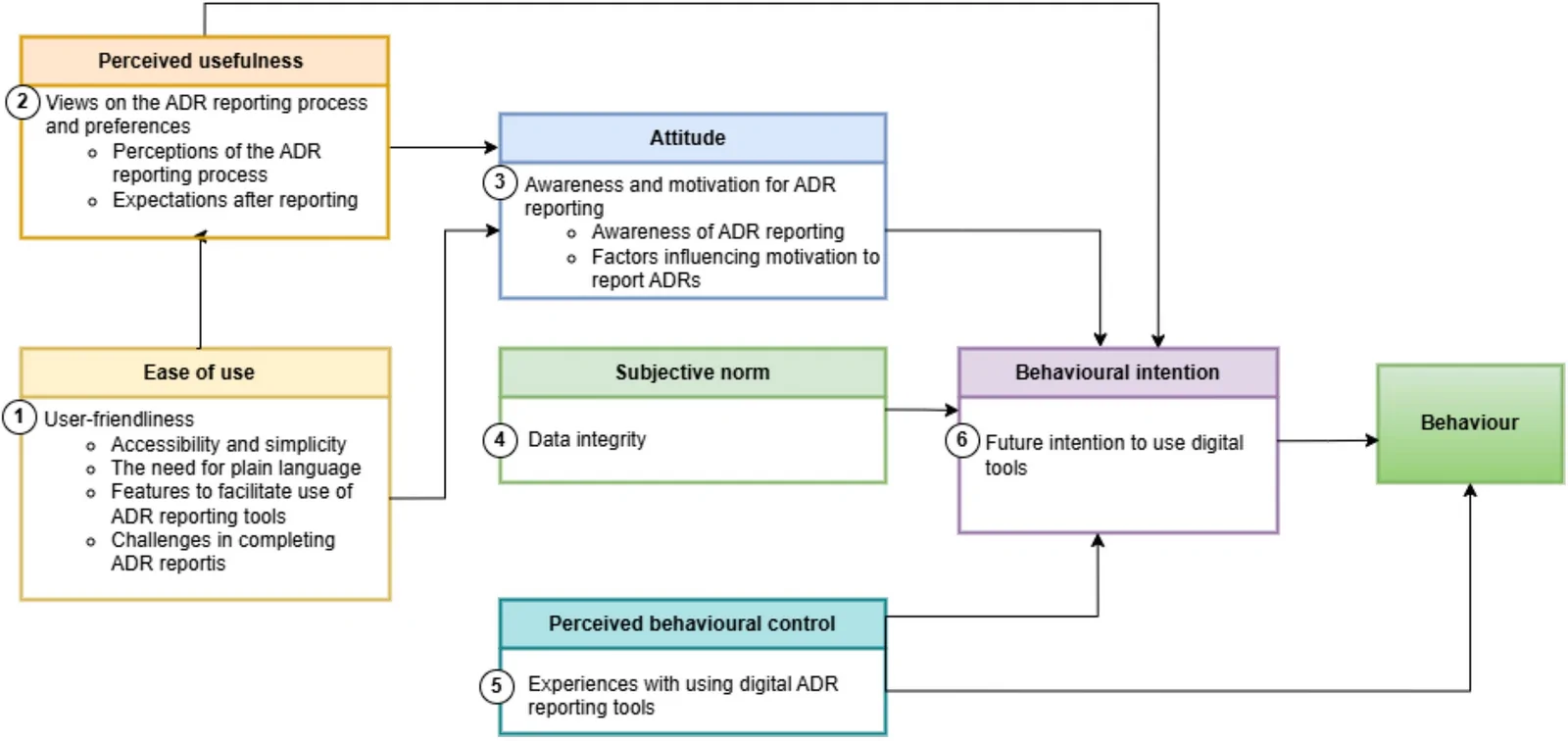
Generated themes and sub-themes mapped into the framework of C-TAM-TPB [32]

The findings of the interviews resulted in the generation of six themes, three of which included sub-themes. Mapping of these themes and sub-themes into the six domains of the C-TAM-TPB is shown in Fig. 1.

### Ease of use

#### User-friendliness

##### Accessibility and simplicity

This sub-theme focused on the overall usability of digital ADR reporting tools, including navigation, layout, and the use of clear and accessible terms to support ease of use. Participants emphasised the importance of a digital tool that is straightforward, uncomplicated, and readily accessible for generating ADR reports. Participants highlighted the importance of a form that can be completed quickly. Additionally, consumers highlighted the significance of avoiding the requirement for registration or authentication when submitting an ADR report, as this process could be burdensome.

“If you’re member of the public because you're not getting paid to do this job, you really need to make it as simple as possible […]. […] if I'm a member of the public, doing this out of goodwill, as soon as it becomes difficult, people […] start dropping out of it and not doing it.” (P14)

Consumers appreciated the use of images in ADR reporting forms to help identify medicine details such as the name and registration number, making it easier to understand what to report and where to find the information. They suggested a clear, easy-to-read layout, avoiding excessive text and small fonts. Participants recommended adding descriptions of terms and using consistent, simplified terms. For example, selecting a single term to describe ‘side effects,’ ‘adverse event,’ ‘adverse reaction,’ or ‘symptoms’ rather than using them interchangeably, as this could be confusing.

“Images would also make it easier for someone.” (P5)

“I think it's fine for healthcare professionals for a doctor or a pharmacist reporting it or something like that. But I think for consumer […] there are some technical terms in there.” (P12)

Participants expressed that simplicity and accessibility were key factors in their preference for choosing websites or apps for ADR reporting. Some preferred websites due to the large amount of information required for ADR reports, with accessibility of computers, ease of entering text, intuitive navigation, and better screen readability identified as key advantages for reporting via computer rather than via an app. Others expressed a preference for apps, suggesting their ease of use, convenience, accessibility, and portability as primary reasons.

“[…] most of my apps are on the tablet and the phone and so it's a smaller screen and […] I find it harder to work with and if I'm completing any forms […], I do them preferably on the computer via the website.” (P10)

“A phone app is probably more convenient. That's all because […] you can do it wherever you are. You'll usually have your phone with you.” (P4)

##### The need for plain language

This sub-theme specifically reflected participants’ difficulties in understanding medical terminology and their preference for consumer-friendly, lay language within ADR reporting tools. Participants identified that the use of medical terminologies is a barrier affecting the user-friendliness of ADR reporting tools. They suggested that the language used in ADR reporting tools should be comprehensible to individuals with limited health literacy. Specifically, participants proposed the inclusion of alternative terminologies or definitions for complex medical terms in parentheses to enhance consumer understanding.

“My only concern is that the language that is used in all of these sort of reporting documents is too sophisticated for many people.” (P4)

For example, participants expressed experiencing confusion regarding the phrase “route of administration” and associated options such as ophthalmic, auricular, intramuscular, intravenous, transdermal and subcutaneous. It was suggested that descriptions be provided in brackets using clear, layperson-friendly language. Participants also recommended options like “I don’t know” or “none” to allow them to proceed without interruption and reduce reporting burden. Participants indicated challenges in understanding questions related to outcomes, particularly terms like “recovered/resolved with sequelae,” which was perceived as complex.

“The terminology and the medical jargon, that is very familiar to a medical professional, but me as a consumer it's unfamiliar, so it makes me feel like this isn't designed to hear me.” (P11)

##### Features to facilitate use of ADR reporting tools

Participants suggested several features that they believed would facilitate ADR reporting using a digital tool. These features include clear directions on what is required for reporting, pre-populated fields for repeated information, and tips to clarify what is required for each question. Participants also suggested additional features like a diagram at the beginning of the form showing the overall reporting process, to facilitate understanding.

“The diagram of the process is the strategy to use so you know at the beginning rather than having to scroll through an entire document is what's required.” (P1)

Participants had differing preferences regarding how to report names of their medicines. Some preferred a free-text space to write the names manually, while others favoured a dropdown list of medicines for selection.

“There's so many drug names that I can only imagine a drop-down menu would be really lengthy.” (P5)

“I think having a drop down would actually be helpful and potentially quicker and also in terms of spelling […] because some medicines […] have got similar names so you could misspell.” (P8)

Participants suggested having an ADR reporting tool tailored for consumers, rather than reusing the same form developed for HCPs. They recommended a feature that inquires whether the individual reporting is a consumer or a HCP at the start of the ADR reporting tool, with terminology adjusted accordingly to accommodate each group.

“There should be two separate ways of reporting, probably makes it easier. […] Separate ones for healthcare professionals and consumers, and I think it's the most important thing.” (P12)

##### Challenges in completing ADR reports

Participants identified that making questions compulsory is a barrier that could prevent consumers from completing ADR reports. While some said they would still finish the form by choosing suitable options, others felt mandatory questions might cause consumers to become stuck, potentially leading them to stop the reporting process. Some participants suggested that questions should not be compulsory and if required, further details could be obtained later through follow-up.

“Where those compulsory boxes are with […] the Asterix, if the options aren't super-duper clear, then you run the risk of the person stopping there and not even submitting the form.” (P6)

### Perceived usefulness

#### Views on the ADR reporting process and preferences

##### Perceptions of the ADR reporting process

Participants emphasised the significance of integrating ADR reporting tools with existing patient health records to facilitate reporting. They suggested that given most patient information is already documented within these systems it would make completing and submitting ADR reports easier.

“There's a lot of information already captured in government sources like My Health Record, whether some of the report, particularly the […] demographic information, could always be prepopulated.” (P8)

Participants shared their perspectives on submitting ADR reports to both HCPs and the national medicines regulator. Some participants indicated that the ability to submit reports concurrently or forward a summary to their HCPs would simplify system.

“That [would] make it much easier. Simpler than contacting everyone separately, I think. There's, particularly healthcare professionals such as your doctors hard to get an appointment to see a doctor to report something. So that would make it easier if you just forwarded it.” (P12)

##### Expectations after reporting

Participants expressed different expectations following the submission of a report. Some participants indicated that receiving a confirmation email acknowledging submission would be sufficient. Others expressed a preference for a follow-up email providing details on what happened to their report, including whether it was reviewed, if similar reports had been submitted previously, and any relevant information related to their case.

“It doesn't need to be a complicated follow up. But at least that someone's looked at it, that's good to know, not that's necessarily being received by an email box, but someone's looked at, done something.” (P12)

Some participants were unsure of what to expect after submitting an ADR report. Others suggested having options such as registering for a newsletter, receiving medicine safety information, receiving notifications advising patients to consult their GP, or informing the GP responsible for their care about any concerns related to their medicine for further action.

“It might be useful generally if there was something in the automatic response that reminded people to notify their doctor.” (P5)

“If maybe people can register for a newsletter […] that would keep me up to date with the platform.” (P6)

### Attitude

#### Awareness and motivation for ADR reporting

##### Awareness of ADR reporting

While some participants told their HCPs about ADRs they experienced, others did not. Additionally, most participants were unaware that they could report their ADR online directly to the national medicines’ regulator. They indicated that if they had known about such reporting options, they would have been more likely to submit a report.

“There is no clear information about where to go to report those side effects. I'd have to call the drug company or something, it's like you don't wanna just get put on. […] If there was an app or something like that, I would put the information in there, […] you don't have to go to a lot of effort.” (P3)

Some participants with prior experience submitting ADR reports to the national medicines’ regulator noted a lack of feedback or follow-up after submitting their report. They expressed uncertainty regarding whether their report had been received or what actions, if any, had been undertaken and this was a barrier to future reporting.

“There was never any follow up, so it was sort of just, this is the medication, this is the side effect and then there's no follow up, so I don't quite see the point of having recorded it really.” (P12)

Participants suggested several strategies to enhance awareness of ADR reporting to the medicine’s regulator using an online platform, including advertising through social media, television, newsletters, and distributing flyers and posters in hospital and clinic reception areas to inform consumers.

“Perhaps there needs to be something regularly on the social media. I don't know if I've ever seen anything at the medical centre advertising […] online adverse event reporting, so obviously specialists and doctors are prescribing and vaccinating. There needs to be something visible in their reception areas about you know had an adverse effect, here's how you can report it.” (P10)

##### Factors influencing motivation to report ADRs

Participants identified factors that motivated them to report ADRs, as well as barriers that deterred them. Consumers reported that the severity or rarity of the ADR, the necessity for documentation of the ADR in their medical records, the impact of the ADR on their life, the potential danger to others and feeling unheard by their HCPs were motivating factors for reporting.

“I told him [general practitioner] what had happened so that I was never given that particular [medicine] again.” (P4)

“I felt like I wanted to pass on my experience to others, to warn them.” (P11)

“I've had mixed reactions from doctors as to whether, it was or wasn't a side effect, but I felt clearly that it was.” (P7)

In contrast, participants described reasons for not reporting ADRs that included the ADR being already known, lacking sufficient information to make the report, and the belief that it was the responsibility of their HCP to report the ADR.

“Because it was a known side effect, I didn't feel as if I had to tell anyone.” (P14)

“I think this reporting should be done by the doctor.” (P13)

### Subjective norm

#### Data integrity

Participants described what makes them trust using digital ADR reporting tools and their privacy and security concerns of providing their personal information. Participants encouraged the use of digital ADR reporting tools, especially if linked with existing electronic patient health records.

“If you were using a website that you knew was secure like the [government] website. You might have more trust in that form, whereas if it was another website I mean, it may be secure, but people need reassurance that their information whether it's anonymous or not is going to be secure.” (P8)

Some participants indicated that they would not be concerned about their privacy and the security of their information when reporting ADRs online to the regulator. However, other participants sought assurance that their sensitive information would remain confidential, particularly when using non-government websites.

“You […] need to know that the information that you're sending the [regulator] or to your GP is being treated in confidence and it's not going to be passed on to other parties.” (P4)

### Perceived behavioural control

#### Experiences with using digital ADR reporting tools

This theme captures participants’ experiences both prior to and during interaction with the example online ADR reporting tool presented in the interview. Participants’ perceptions of the ease or difficulty of use varied. Some stated that the form was easy to complete, while others noted that it required a lot of information, which could be time-consuming and difficult for the general public. They recommended collecting pertinent information initially, with follow-up for additional information if needed.

“It was simple […] filling out the form.” (P12)

“It would be off putting to someone who didn't want to spend 20 minutes to half an hour submitting the form.” (P10)

“I think as it is currently it's more on the difficult side.” (P6)

## Behavioural intention

### Future intention to use digital tools

Many participants expressed a willingness to use online ADR reporting tools in the future, particularly if they encounter new ADRs. However, participants emphasised that any ADR reporting tool designed for consumers should be simple and easy to understand.

“Now I know that it's available I would use it, but I didn't even know that there was the facility to do this.” (P4)

“It's only by reporting adverse events that those things are compiled into useful information for the general community. So, I see it as a responsibility for myself as a consumer to interact, […], to seek out this sort of thing and to offer the experience I've had in order to share that in case it might be useful.” (P11)

## Discussion

We found that while consumers indicated a willingness to use online ADR reporting tools to report ADRs, they were largely unaware of the existence of such tools. Consumers want online ADR reporting tools that are user-friendly, accessible, specifically designed for consumers using minimal medical jargon, clear direction on reporting requirements, a tool written in lay language, integrated with patient health records, that allow one report to be shared with both HCPs and regulators.

Our findings align with previous studies indicating that user-friendly interfaces which reduce complexity in the reporting process, encourage higher participation rates among HCPs and consumers [20, 33–36]. Similarly, consistent with our findings, a prior study conducted in Thailand suggested that consumer reporting forms should differ from those used by HCPs and be written in language accessible to consumers with limited health literacy [17]. A study covering ADR reporting from 2015 to 2019 indicated that countries with separate ADR reporting forms for consumers and HCPs like the Netherlands (58%), Denmark (38%), Belgium (45%), and the UK (18%) had higher consumer reporting rates than those using the same forms, such as Canada (14%), Taiwan (0.1%), and the Philippines (0.03%) [18]. Currently in Australia, the medicines regulator provides a separate pathway for consumers to report ADRs; however, it uses the same form for all reporter types [37, 38]. Our findings suggest that consumers would prefer a separate online reporting form specifically designed for them.

Barriers to using online ADR reporting tools identified in our study include unawareness that consumers can report ADRs to the regulator, the need to register or log in, lengthy reporting forms, cluttered layouts with excessive text and small font, the use of medical terminology, compulsory questions, and the absence of feedback or follow-up after submitting a report. These findings are consistent with previous studies reporting similar barriers [17, 33, 39]. This underscores the need for a reporting form specifically designed for consumers, using minimal jargon, providing clear explanations of the information being requested, and avoiding compulsory questions where possible.

Our findings provide support for the relevance of the C-TAM-TPB framework in understanding factors influencing consumers’ behavioural intentions toward online ADR reporting tools. Specifically, perceived ease of use was an important factor. Ease of use in turn, influenced both perceived usefulness and attitudes toward using online ADR reporting tools. Participants indicated that perceived usefulness would increase if they received acknowledgment and feedback from the regulator, and that awareness of the tool contributed to a more positive attitude and willingness to report ADRs. The role of subjective norms was also evident, as participants expressed trust in the online ADR reporting tool when integrated with their existing patient health records, consistent with prior studies indicating successful integration of patient-generated data into electronic medical records and health apps [40, 41]. However, concerns about confidentiality and data privacy highlighted the need to address perceived risks associated with information sharing. In line with the perceived behavioural control construct, participants described varied experiences using online ADR reporting tools, suggesting that simplifying design and tailoring it for consumers would enhance usability and strengthen behavioural intention. Overall, our findings confirm that consumers’ behavioural intention to use online ADR reporting tools is high, which may translate into increased use if identified barriers are addressed. This highlights the need to improve consumer awareness of ADR reporting systems and to enhance digital tools through consumer-centred design, including plain language, simplified forms, visual aids, and clear guidance on reporting requirements. These findings also have important implications for older adults, culturally and linguistically diverse communities, and individuals with lower health literacy, who may face additional challenges in accessing and using digital ADR reporting tools. Future research and online ADR reporting tool development should focus on improving perceived ease of use, strengthening trust through integration with health records, and co-designing the online ADR reporting tool with consumers to address barriers related to language and privacy.

A strength of this study lies in its methodological rigour, achieved via analyst triangulation, detailed contextual descriptions, inclusion of diverse participants’ characteristics, a transparent audit trail, and team debriefing to ensure credibility, transferability, dependability, and confirmability. One important limitation of this study is that the online nature of the participant recruitment method may have resulted in the underrepresentation of individuals who do not use electronic devices, limiting the transferability of the findings. Consumers with limited digital literacy or access may have different experiences, needs and barriers to using digital ADR reporting tools. Additional limitations include potential self-selection bias, whereby individuals with greater interest in the study topic may have been more likely to participate, potentially affecting the diversity of perspectives captured. Therefore, interpretation of the results should take these limitations into account.

## Conclusion

Consumers indicated a willingness to use online ADR reporting tools to report ADRs. User-friendly design, visual aids, integration with patient health records, and reporting forms tailored to consumers using lay language were identified as potential facilitators to enhance consumer engagement. Conversely, barriers such as lack of awareness, login requirements, medical jargon, compulsory questions, and the absence of feedback may impede their use. These findings provide insight into factors that may influence consumer engagement with online ADR reporting tools and may inform future development of more user-centred pharmacovigilance systems.

## Supplementary Information

Below is the link to the electronic supplementary material.

Supplementary file1 (DOCX 46 KB)

## Data Availability

The data that supports the findings of this study are available from the corresponding author upon reasonable request.
