# Supplementary material for: Consumers’ experiences and perspectives on reporting adverse drug reactions using digital tools: a qualitative study
Source: Int J Clin Pharm. 2026 Jul 22;48(5):2094–104. doi: 10.1007/s11096-026-02193-1 (PMC13569527; doi:10.1007/s11096-026-02193-1)
Supplement: Supplementary file 1 [file 11096_2026_2193_MOESM1_ESM.docx]

**Supplementary File: Interview Guide**

**Questions for semi-structured interviews**

**Demographics**

- Can I ask you what age range you fall into, would that be 18 to 44, 45 to 64, 65 or higher?
- May I also ask your highest level of education? *(free response from participant – if direction or clarification is required, prompt with: postgraduate degree, bachelor’s degree, secondary school completed, primary school completed etc).*

**Part I – Consumers’ experience with side effect reporting and the barriers and enablers to side effect reporting.**

1. Can you tell me about a time you experienced a side effect/s? —describe what happened?

Probes:

a. Did you discontinue the medicine or take treatment for the side effect or any consequences?
b. How many times did you experience the side effect/s?
c. Did you report any of the side effect/s you experienced?
I. If you have not reported it, why did you not report it?
II. If you have reported it, to whom did you report it? (*probe*: What made you report it?
i. What reporting method did you use?
ii. Why did you choose the method you used for reporting?
iii. If you have experienced any challenge(s) during the reporting process, please describe what they were?

**Part II – Familiarity and experience of consumers about the Therapeutic Goods Administration (TGA) online side effect reporting website.**

2. Do you know the Therapeutic Goods Administration (TGA) (the medicine regulatory agency of the Australian Government) has an online side effect reporting website?

Probes:

a. Have you ever used it for reporting side effects?

If you haven’t used it, why did you not use it?

If you have used it, why did you decide to use it?

b. *The screenshot of the Therapeutic Goods Administration online side effect reporting form will be displayed for the participants, and they will be asked if they can understand and complete the information requested in the Therapeutic Goods Administration online form.*

*Followed by the questions:*

How easy/difficult do you think this online form would be to complete? Why?

i. How logical did you find the structure of the process?
ii. What parts did you find difficult to complete?
iii. What made it difficult for you? (probe: is it the terminology in the form? Was the language clear and easy to understand?)
iv. Did you know how to answer all of the questions in the form – if no, can you tell me the questions you don’t know how to answer in the form? What about these questions made them hard for you to answer (probe: terminology? Way it was worded? Technical language?)
    - What do you think you would do if you were completing the form and came to a question like this that you weren’t sure how to answer (prompts: Do you leave it blank and move to the next question? Or Stop completing the form altogether?)
c. Is there anything that you would improve about the Therapeutic Goods Administration online side effect reporting website? (probe if yes: what would that be?)
d. How likely is it that you will use this platform in the future?

**Part III – Consumers' views on developing a digital tool for reporting the side effects of medicines.**

3. Can you tell me about your experience of using a digital tool for other health-related activities, for example, have you ever used the HotDoc app, or website to book an appointment with your doctor? What do you find easy to use about them?

Probes:

a. What other digital tool did you use for health care activities? What do you like/dislike about them?
b. Do you prefer to use a mobile app or a web-based system to get information or to do an online activity? Why?
c. Can I ask, what you would do if you experienced side effects from medicines, would you choose to inform your healthcare professional or report to the TGA or you would tell both to the HCP and TGA? Why?
d. If a digital tool offers the option of sending your side effects of medicine to both your healthcare professional (HCP) and the Therapeutic Goods Administration (TGA), what would be your thoughts on using it?
I. If you don’t support using digital tools to send it to both HCP and TGA, what are your reasons?
II. If you support using it:
    i. Would you like to submit the same data to both your HCP and TGA simultaneously, or would you prefer to send separate data based on the information you wish to submit to each body?
    ii. Do you anticipate receiving a response from your HCP and/or the TGA?
    iii. Can you describe the responses you would expect from your HCP and/or the TGA?
    iv. Will the kind of responses you receive from the reporting body affect your likelihood of reporting side effects in the future? How?
e. What do you think of linking side-effect reporting digital tools with existing medication management apps, for example, MedAdvisor or other medication management apps? How will this impact your side-effect reporting practice?
f. Is there anything that you think is important for us to consider while developing a digital tool for reporting side effects?

**Thank you very much for taking the time to participate in this interview.**
